# Totally intracorporeal colorectal anastomosis (TICA) after segmental colorectal resection for deep endometriosis: technical notes and case series

**DOI:** 10.1007/s00404-025-08040-4

**Published:** 2025-05-06

**Authors:** Francesco Santullo, Alessandra De Cicco Nardone, Miriam Attalla El Halabieh, Claudio Lodoli, Carlo Abatini, Federica Ferracci, Federica Campolo, Greta Benvenga, Giovanni Scambia, Fabio Pacelli, Manuel Maria Ianieri

**Affiliations:** 1 Surgical Unit of Peritoneum and Retroperitoneum, Fondazione Policlinico Universitario A. Gemelli, IRCCS, Largo Agostino Gemelli 8, 00168 Rome, Italy; 2 Unit of Oncological Gynaecology, Women’s Children’s and Public Health Department, Fondazione Policlinico Universitario Agostino Gemelli, IRCCS, Rome, Italy; 3 Catholic University of the Sacred Heart, Rome, Italy

**Keywords:** Deep endometriosis, Colorectal resection, Colorectal anastomosis, Laparoscopic surgery

## Abstract

**Purpose:**

The aim of this study was to evaluate the safety and feasibility of totally intracorporeal colorectal anastomosis (TICA) in patients undergoing colorectal resection for the treatment of deep endometriosis (DE) affecting the bowel.

**Methods:**

Between January 2021 and August 2024, 33 consecutive patients with DE treated with segmental colorectal resection were enrolled. In 30 patients, TICA was performed. Demographic, operative, and postoperative data were collected retrospectively.

**Results:**

The mean distance between the endometriotic nodule and the anal verge was 11.5 (7–18) cm. The mean operative time was 282.83 (190–512) minutes. No major intraoperative complications occurred. Three (10%) patients developed a minor (Clavien‒Dindo grade I/II) postoperative complication.

**Conclusion:**

TICA is a safe and feasible technique and represents a valid alternative reconstruction method after colorectal resection for DE.

## Introduction

Deep endometriosis (DE) is an infiltrative form of endometriosis affecting the bowel, usually the rectum and sigmoid colon, in 8–12% of cases [1, 2]. Colorectal resection is the most radical surgical approach in the treatment of selected cases of DE affecting the bowel [3]. However, mini laparotomy in combination with colorectal resections is associated with postoperative complications, such as wound infections, incisional hernias, and worse cosmetic results.

Hence, over the years, the mainstream surgical treatment of DE has been to reduce surgical trauma and invasiveness, developing new anastomotic techniques, such as natural orifice specimen extraction (NOSE) [4, 5]. Recently, we described a new technique of totally intracorporeal colorectal anastomosis (TICA) [6] after colorectal resection for DE, also used successfully for some cases of recurrent ovarian cancer [7].

The aim of this study was to evaluate the safety and feasibility of TICA in patients with DE in terms of the short-term outcome.

## Materials and methods

**Fig. 1:**
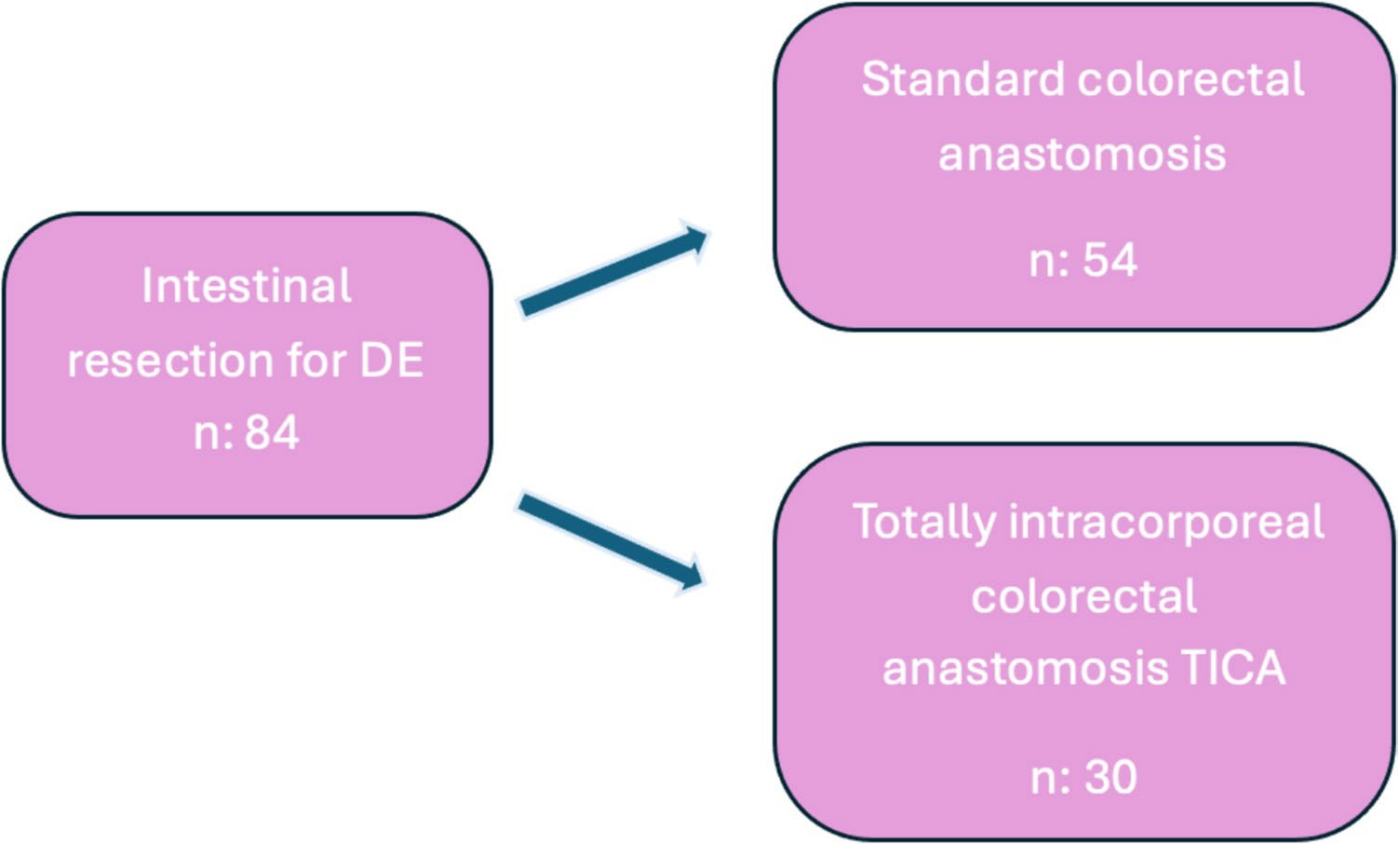
This flow diagram illustrates the total number of patients who underwent surgery for deep endometriosis (DE) at our center from 2021 to 2024, detailing how many of them underwent segmental intestinal resection and the type of colorectal anastomosis performed

The present paper reports the results from 33 consecutive single-center colorectal resections for DE performed at Fondazione Policlinico Gemelli between January 2021 and August 2024, selected to perform TICA. All patients with intestinal DE were evaluated by a multidisciplinary team, including a gynecologist, a radiologist, and a general surgeon specializing in minimally invasive colorectal surgery. We included all patients who underwent laparoscopic radical surgery for DE with segmental intestinal resection performed with IMA preservation and pelvic nerve-sparing techniques [8] in which the feasibility of performing the TICA was assessed preoperatively; if not feasible, a colorectal anastomosis was performed using the standard technique (Fig. 1). Segmental intestinal resection was performed in patients with symptoms of bowel obstruction, nodules > 3 cm, multiple nodules, full-thickness invasion reaching the mucosa and failure of conservative surgical techniques.

Data regarding history and preoperative evaluation were recorded. All women were subjected to rectovaginal examination, advanced transvaginal ultrasonography and magnetic resonance imaging. In cases of sub-occlusive symptoms, either a colonoscopy or double barium enema was further required to evaluate the stenosis. The operative and postoperative data were reviewed. Complications were subdivided into minor complications, corresponding to Clavien‒Dindo grade I and II, and major complications, corresponding to Clavien‒Dindo grade III and IV [9]. Informed consent was obtained from all individual participants included in the study. The study was approved by the local Institutional Review Board (IRB-number of protocol DIPUSVSP-25-06-2156).

### Preoperative care, surgical technique and postoperative care

**Fig. 2:**
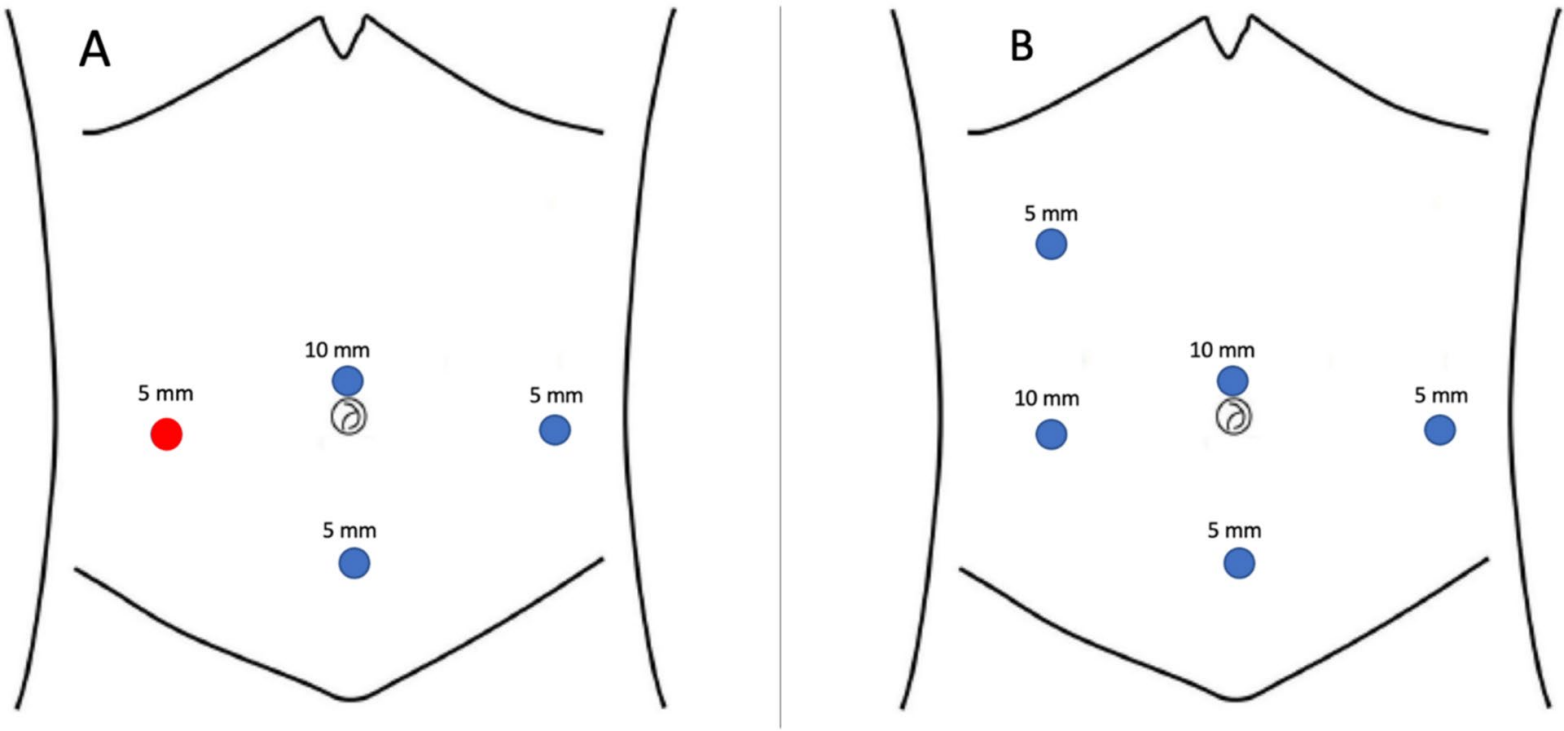
Placement of the surgical ports during gynecological procedures (**A**) and TICA (**B**)

**Fig. 3:**
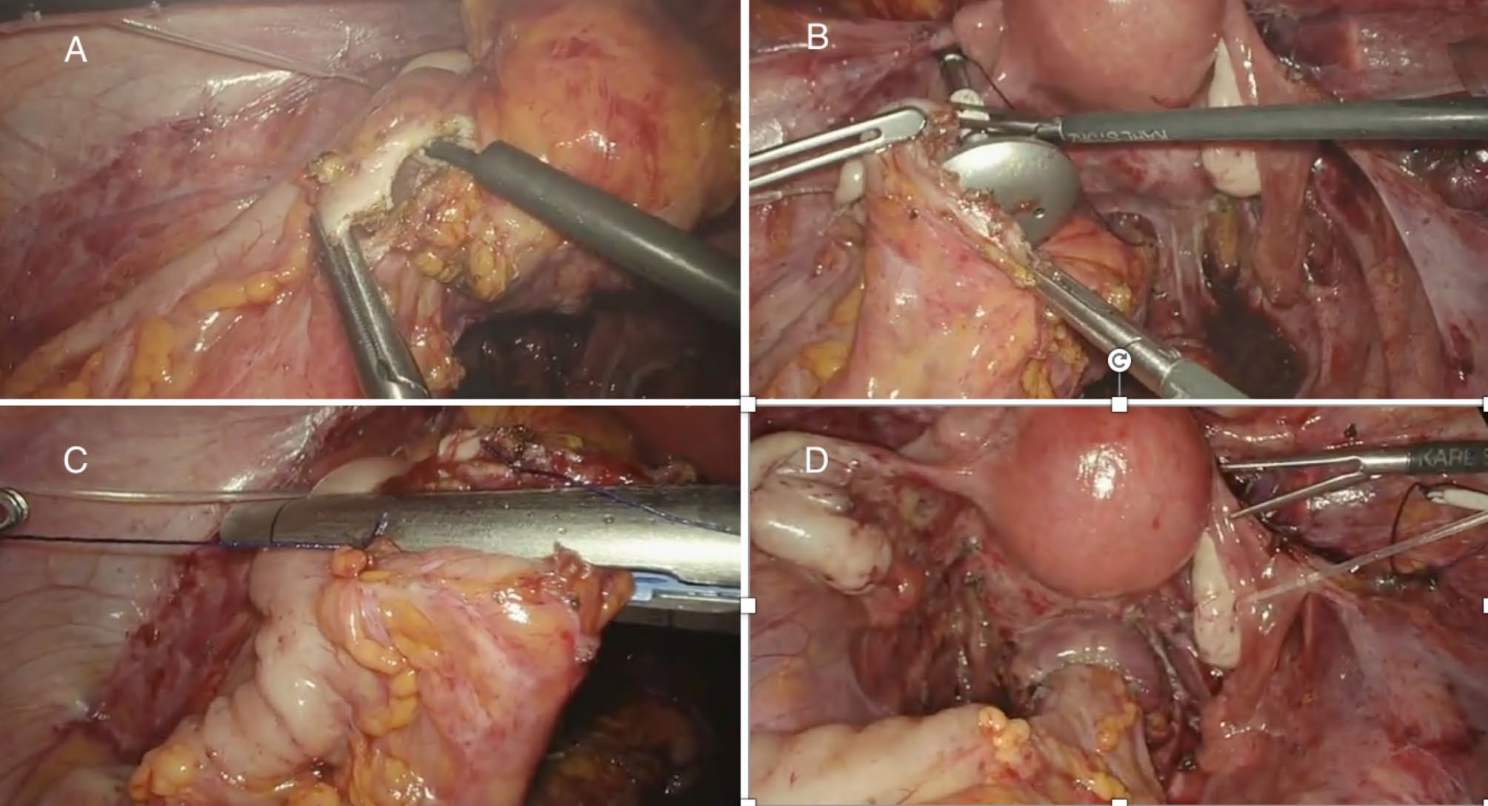
Colotomy is performed at the colonic wall just proximal to the endometriotic nodul (**A**), the anvil is introduced into the colon through the colotomy (**B**), the colon is transected with a linear stapler and the suture is pulled out of the colon (**C**), colorectal end-to-end anastomosis (**D**)

In preparation for surgery, all patients followed a 5-day residue-free diet and underwent mechanical bowel preparation consisting of a 4-l split dose of Macrogol: 2 L 2 days before surgery and 2 L the day before surgery. All patients received antibiotic prophylaxis 30 min preoperatively (1–2-g cefazolin iv. and 500-mg metronidazole iv.). All patients were operated on by a multidisciplinary surgical team widely experienced in laparoscopic surgical excision of bowel endometriosis, including a gynecologist and a colorectal surgeon. The operative room setup was the same for all procedures. The patient was positioned in an anti-Trendelenburg position. Pneumoperitoneum was established using a 12 mm umbilical trocar, and the gynecological surgical procedures were performed using three 5-mm trocars placed in the suprapubic area, left iliac fossa, and right iliac fossa. Intestinal resection was performed using the same trocar positions except for a 12-mm trocar in the right iliac fossa in place of the 5-mm trocar and a fourth 5-mm trocar on the right flank (Fig. 2). The peritoneum of the mesosigma was opened above the root of the inferior mesenteric artery (IMA), staying as close to the bowel wall as possible. The sigmoid vessels, which supply the bowel segment to be resected, were progressively identified and selectively ligated. The dissection was carried out until reaching the rectal wall below the endometriotic nodule, and then the rectum was transected with a linear stapler, Echelon Flex^™^ Endopath^®^ Staplers (EFES) 60 mm (Ethicon, Cincinnati, OH, USA). Before anastomosis, the anvil of a circular stapling device (EEA^™^ circular stapler with Tri-Staple^™^ technology, 28 mm or 31-mm Medium/Thick, Covidien, New Haven, CT, USA) was prepared with a 0 Vicryl suture, which was bound at the hole of the tip. The anvil was brought into the abdominal cavity through the opening for the 12-mm port in the right abdominal flank. A colotomy was performed at the colonic wall just proximal to the endometriotic nodule (Fig. 3). The anvil was introduced into the colon through the colotomy (Fig. 3). The linear stapler was arranged to include the whole colotomy. The suture attached to the rod of the anvil was removed from the superior border of the colotomy, keeping the Vicryl suture out of the linear stapler. The colon was then transected with the linear stapler (Fig. 3). The suture was pulled out of the colon, allowing the rod of the anvil to exit the colon next to the suture line. The circular stapler was introduced in the rectum, and end-to-end anastomosis was performed. The specimen was extracted through the 12 mm port on the right flank. At the end of the procedure, an air leak test was performed to evaluate anastomosis integrity. One drainage was left in place in the pouch of Douglas. On the first postoperative day, oral fluid intake was allowed, and from the second postoperative day, patients started a low-fiber diet until tolerance for solid food and passage of flatus and stool occurred. Postoperative pain was measured by VAS score, and all patients received paracetamol 1 g iv every 8 h on demand and continued orally when feasible.

## Results

Patient demographics and preoperative characteristics are summarized in Table 1. Among the 33 resections, we experienced 3 (9.1%) intraoperative complications that did not allow us to complete the TICA. In one case, during the extraction of the anvil, the tip was caught in the suture line. In the other two cases, the colon was too small, so we could not laparoscopically introduce the anvil. In both cases, we performed a classical anastomosis with the Pfannenstiel incision. Hence, we excluded these three patients, and we conducted the subsequent analysis only on the 30 patients who underwent TICA. The mean patient age was 38.83 (32–52) years, and the mean patient BMI was 21.63 (18–27) kg/m2. The mean distance between the endometriotic nodule and the anal verge was 11.5 (7–18) cm. The median size of the intestinal endometriotic implants was 34.2 (20–40) mm. The mean rASRM score was 61.5. Table 2 summarizes the operative and postoperative details. The mean operative time was 282.83 (190–512) minutes. The mean estimated intraoperative blood loss was 129.16 (50–300) ml. No major intraoperative complications occurred, and no conversions to laparotomy were necessary. Three (10%) patients developed a minor (Clavien‒Dindo grade I/II) postoperative complication: two postoperative bleeding of the anastomosis that did not require any surgical or endoscopic intervention and one postoperative ileus that was medically treated. No major (Clavien‒Dindo grade III/IV) complications occurred. The complete operative details and postoperative complications are shown in Table 2. After 3 months, three patients (10%) with protective ileostomy underwent rectal contrast enema and subsequent ostomy closure.

**Table 1:** Patient demographics and preoperative characteristics

Variables	*N *(30)
Age (mean, SD) years	38.83 (32–52)
BMI (mean, SD)	21.63 (18–27)
Previous surgery for endometriosis	8 (26.7%)
Ormonal Therapy 3 months previous surgery	30 (100%)
Preoperative symptoms	
Dysmenorrhea (vas)^a^	7.58
Dischezia (vas)^a^	5.75
Dysuria (vas)^a^	1.33
Dyspareunia (vas)^a^	6
Chronic pelvic pain (vas)^a^	6.25
Constipation	12 (40%)
Diarrhea	0
Rectal bleeding	2 (6.7%)
Symptomatic colorectal stenosis	13 (43.3%)
STAGE rASRM	61.5
Intestinal DE	
Intestinal lesions > 3 cm	25 (83.3%)
Multiple intestinal lesions	5 (16.7%)
Major nodule dimension (mean, SD) mm	34.2 (20–40)
Nodule location	
Sigmoid colon	13 (43.3%)
Rectum	17 (56.7%)
Distance from anal verge (cm)	11.5 (7–18)

Nominal variables are described with number of cases (*n*) and percent (%)

^a^Median value

**Table 2:** Intraoperative and posoperative data

Variables	*N* (30)
Intraoperative	
Blood loss (mean, SD), ml	129.16 (50–300)
Operative time (mean, SD) min	282.83 (190–512)
Diverting enterostomy%	3 (10%)^a^
Intraoperative complications	0
Conversion, *n* (%)	0
Postoperative	
Start of postoperative diet	Day 1 23 (76.7%) Day 2 7 (23.3%)
Time to resume intestinal function (days)	3.5 (1–6)
Postoperative hospital stays days	5.4 (4–10)
Postoperative complications (Clavien–Dindo)	3 (10%)
Grade I–II	3 (10%)
Grade III–IV	0
Readmission, *n* (%)	0

Nominal variables are described with number of cases (*n*) and percent (%)

^a^One patient with simultaneously ureteral resection and reimplantation, one patient with simultaneously ileocecal resection, one patient with multiple comorbidities

## Discussion

We present a series of thirty consecutive colorectal resections for deep endometriosis (DE) performed with a nerve-sparing technique plus IMA preservation [10] in which TICA was successfully performed without any modification of the standard practice. The 30-day complication rate is similar to that reported in the literature by other authors [11, 12] after colorectal resections for DE, showing that TICA is a safe, effective and reproducible anastomotic surgical technique.

**Fig. 4:**
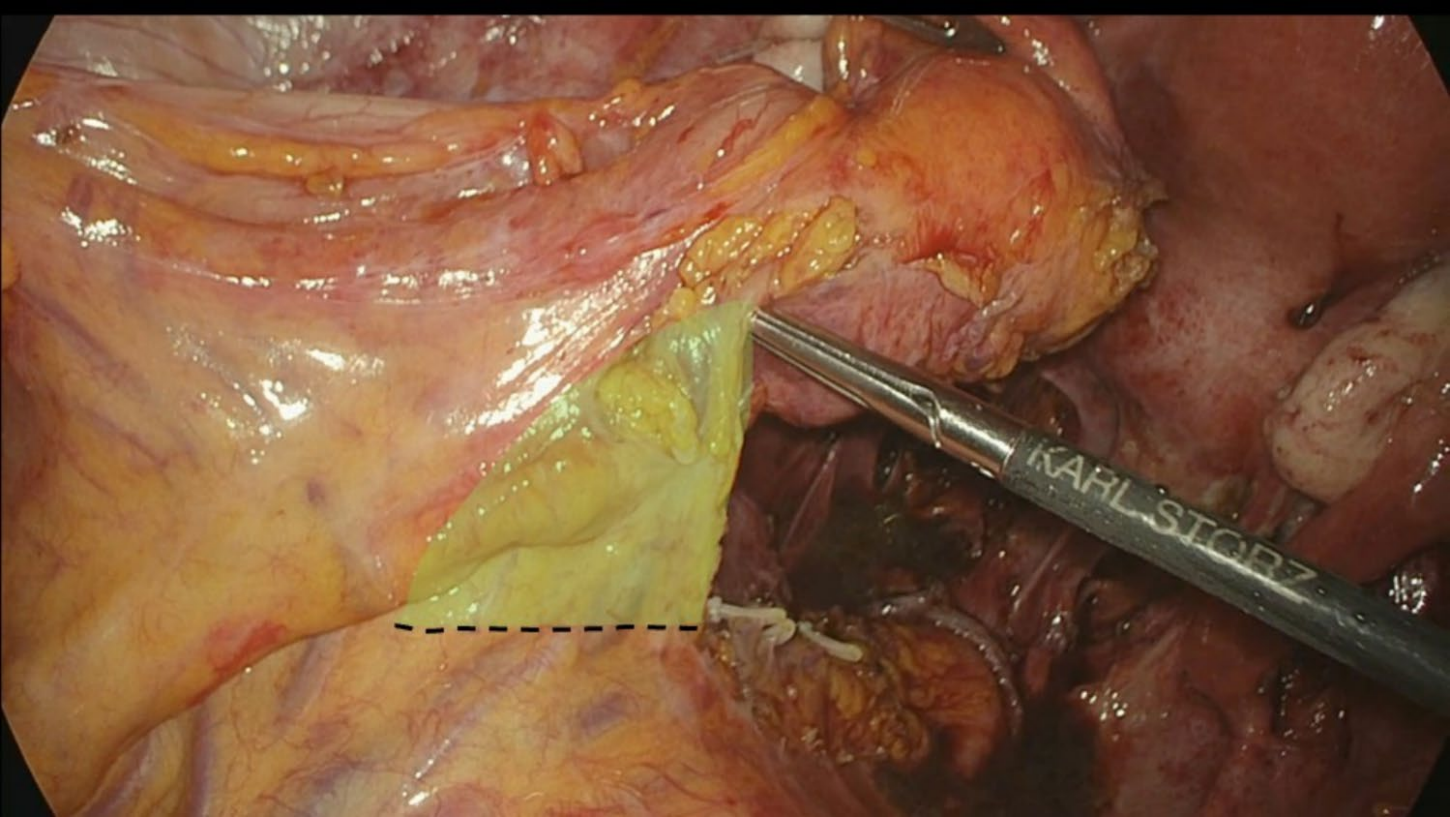
Shown here is the part of mesocolon and surrounding sigmoid vessels and nerves saved performing a TICA

The concept behind this technique’s design is that in most cases, only a short tract of the bowel is resected during DE colorectal surgery; moreover, there is no need to resect the mesum as in oncologic colorectal surgery. In our experience, after a typical bowel resection for DE, a large part of the mesum should be resected only to remove the colon from the Pfannenstiel incision (Fig. 4). When performing a TICA, there is no need to achieve wide mobilization; therefore, a considerable part of the mesocolon and the surrounding sigmoid vessels and nerves are saved.

Considering this concept, a better indication for TICA is when intestinal DE appears as a single nodule or even multiple but closely spaced nodules, and a short resection can be performed. In contrast, the presence of multiple and outlying nodules, which require a wider resection and mobilization, makes patients less suitable for a safe application of this technique.

In the surgical approach of young women with intestinal DE, our anastomotic technique offers different advantages. First, by avoiding the Pfannenstiel incision, TICA provides better cosmetic results, less postoperative pain and a lower risk of developing postoperative wound infection and post- incisional hernia. Second, there is no need to widen the distal stump resection, as in the case of transanal extraction of the specimen in which the rectal stump is left open at first to extract the specimen and insert the anvil and then resected a second time. This advantage is not relevant in the case of a higher resection but is paramount when a resection in the middle and low rectum has been performed. Moreover, avoiding the opening of the vagina avoids injury to a healthy organ and reduces the risk of rectovaginal fistulas. Another possible advantage of TICA is that performing colorectal anastomosis in a totally intracorporeal way allows us to avoid wide mobilization of the colon, which is normally removed through the Pfannenstiel incision; therefore, a considerable part of the mesocolon and the surrounding sigmoid vessels and nerves are preserved, further reducing the risk of intestinal denervation of the proximal and distal stump. Improvement in functional outcomes should be confirmed by further studies.

Over the years, various laparoscopic techniques of intracorporeal colorectal anastomosis have been developed to improve the minimally invasive approach of laparoscopic surgery in DE [13–15]. The NOSE technique for segmental colorectal resection in patients with DE is one of the most described techniques in the literature due to the outstanding cosmetic results and with a lower risk of incisional hernia and postoperative infections, less postoperative pain, and less analgesia requirements, resulting in a shorter hospital stay [4]. While TICA is similar in many postoperative benefits, the NOSE technique, on the other hand, is burdened with technical surgical complexity [16], leading to complications such as a higher risk of peritoneal contamination from the open bowel [17] and distal rectal stump injury [18] in cases of transanal specimen extraction or rectovaginal fistula when transvaginal extraction is performed [19].

A possible technical concern of our technique is the presence of multiple intersections of staple lines on the circular anastomotic plane. The classical Knight–Griffen double stapling technique (DST) [20, 21] provides two lateral intersecting staple lines (“dog-ears”) and two intersection points with the circular stapling line. Historically, these are considered weak points in DST anastomosis. In TICA, there could be a possible doubling of these intersections because there are two linear stapler lines (proximal colon and rectal stump) that could intersect the circular stapling line. Therefore, there could be up to 4 intersecting points compared to the 2 points of the DST. To avoid this drawback, in some cases, we used 31-mm circular staplers: the wider area of the circular stapler could include one or both extremities of the proximal and, rarely, distal linear stapler, avoiding dog-ear creation. In some cases, we adopted another precaution of an intracorporeal reinforcement suture with 3–0 Vicryl at the intersection point. However, in our experience with colorectal anastomosis with DST, “dog-ears” are not a major risk factor for anastomotic leakage, and there was no case of anastomotic leakage in this case series. We have to report just on case of sub-clinical leakage in one of the patients with protective ileostomy in which the leakage was discovered three months after surgery during the contrast enema. Moreover, this technique involves the preservation of the IMA and the sigmoid vessels to improve the vascularization of both the proximal stump and the distal stump. The downside is that we experienced anastomotic bleeding in 2 patients (6.7%); hence, attention should be given to this specific postoperative complication. The intra-abdominal opening of the colon is part of TICA, and this can lead to possible intra-abdominal contamination, as in NOSE with transrectal extraction. However, in our series, we did not observe an increase in intra-abdominal infections or abscesses. Last, it must be said that TICA is a technically complex procedure requiring advanced laparoscopic skills. The most difficult part of the procedure is introducing the anvil into the proximal colonic stump. In fact, we report 2 cases (6.7%) in which we were unable to complete the procedure, and it was due to problems in introducing the anvil into the proximal stump. However, no differences were seen in terms of in terms of intra and postoperative complications between TICA technique and a classical technique of bowel resection for DE [22].

## Conclusion

Our study shows that TICA is a safe and feasible technique in patients who undergo laparoscopic colorectal resection for DE. Further studies are still required to evaluate a possible improvement in functional outcomes.

## Data Availability

No datasets were generated or analysed during the current study.
